# Effects of various treatments for preventing oral mucositis in cancer patients: A network meta-analysis

**DOI:** 10.1371/journal.pone.0278102

**Published:** 2022-12-08

**Authors:** Tzu-Rong Peng, Fang-Pei Tsai, Ta-Wei Wu

**Affiliations:** Department of Pharmacy, Taipei Tzu Chi Hospital, Buddhist Tzu Chi Medical Foundation, New Taipei City, Taiwan; Siksha O Anusandhan University School of Pharmaceutical Sciences, INDIA

## Abstract

**Background:**

Oral mucositis (OM) is a common side effect of chemotherapy and radiotherapy in patients with cancers. The prevention or treatment of OM in cancer patients is crucial in the treatment of cancer.

**Methods:**

We searched PubMed, Embase, and Cochrane Library for the randomized control trials (RCTs) of interventions for preventing and treating OM. Network meta-analysis (NMA) was performed to estimate odds ratios (ORs) and 95% confidence intervals (CI) from both direct and indirect evidence. The prespecified primary efficacy outcome was the treatment effect of moderate to severe oral mucositis with 12 interventions. The outcome was moderate to a severe grade of OM.

**Results:**

This study included 55 RCTs with 3,552 participants. The results showed that honey significantly lowered the risk of chemo/radiotherapy-induced moderate to severe oral mucositis than placebo (OR: 0.01, 95%CI 0.00 to 0.45), followed by lignocaine (OR: 0.07, 95%CI 0.00 to 0.95). The surface under cumulative ranking curve (SUCRA) values for honey were 0.95, followed by lignocaine (SUCRA, 0.81) and benzydamine (SUCRA, 0.78).

**Conclusions:**

The honey is effective for patients with cancer undergoing chemotherapy or radiotherapy-induced oral mucositis.

## Introduction

Chemotherapy and radiotherapy are the most methods for treating cancer, they can result in serious adverse reactions [1]. Oral mucositis (OM) is one of the main side effects of chemotherapy and radiotherapy, and the incidence rate is 40% to 100%. The incidence of OM is related to age, tumor type, treatment methods, nutritional status, and oral hygiene [2–4]. The symptom of OM is erythema, which can progress to painful ulcerations. Ulcerations in oral mucositis are painful and require local analgesics, which may cause the patient to have difficulty eating and cause malnutrition. Malnutrition status will affect the quality of life of patients and delay chemical therapy and radiotherapy.

The prevention and treatment of OM caused by chemotherapy or radiotherapy remain challenging. Several interventions have been investigated for the prevention and treatment of OM, such as chlorhexidine, benzydamine, sucralfate, povidone-iodine, glutamine, and honey, which have been found to prevent mucositis or reduce the severity of mucositis [5–8]. However, no approach has been completely successful for OM. Therefore, the prevention or treatment of OM remains to be resolved.

Although several meta-analyses have been conducted independently to assess the effects of the different interventions compared with placebo [8–11]. The evidence of meta-analysis was limited due to the lack of multiple comparisons. Network meta-analysis is a methodology for assessing multiple interventions through direct and indirect comparison [12]. Therefore, we performed a network meta-analysis to comprehensively compare and rank the efficacy of interventions used for preventing and treating OM in cancer patients receiving chemotherapy and radiotherapy.

## Materials and methods

### Systematic literature review

This network meta-analysis was performed by the Preferred Reporting Items for Systematic reviews and Meta-Analyses (PRISMA) Extension Statement for Reporting of Systematic Reviews Incorporating Network Meta-analyses of Health Care Interventions (PRISMA-NMA) [13]. We searched the PubMed, Embase, and Cochrane Library database up to 30^th^ December 2021. Titles and abstracts were screened, and relevant articles were independently and fully reviewed by two reviewers (TW Wu and TR Peng). Disagreements were resolved by consensus. No language restrictions were imposed. In the event of duplicate publications, we selected the publication that reported the data of interest most completely. The references of included studies were additionally screened to identify relevant RCTs.

### Study selection and outcome measures

This study was performed by Cochrane Collaboration guidelines [14]. The following information was extracted: author, year of publication, study design, number of enrolled patients, cancer types, prevent or treatment OM, chemotherapy- or radiation therapy-induced OM, and clinical efficacy (the incidence of moderate-severe OM). Trials that met the following criteria were included: (1) randomized control trial, (2) comparison of application between the prophylactic or treatment groups and control groups of patients with cancer with chemotherapy- or radiation therapy-induced OM, (3) included all cancer types, and (4) studies that mentioned patient inclusion and exclusion criteria, mucositis grades, and treatment procedures for all groups. In addition, OM grades were determined using the Radiation Therapy Oncology Group criteria [15], Organization WH. World Health Organization (WHO) handbook for the report [16], or Common Terminology Criteria for Adverse Events [17]. The outcome is presented as the overall odds ratios for the occurrence of moderate-severe OM induced by chemo/radiotherapy in patients with cancer. Severe OM is defined as grades 3–4, and moderate OM as grades 2.

### Data extraction and quality assessment

The Cochrane Collaboration tool was used to assess the risk of bias [14], which covers the potential sources of bias including selection bias (random sequence generation and allocation concealment), performance bias (blinding of participants and personnel), detection bias (blinding of outcome assessment), attrition bias (incomplete outcome data), and reporting bias (selective reporting). Each study was categorized as having either low risk (green), unclear risk (yellow), or high risk (red) of bias. The risk of attrition bias was considered to be low if the dropout rate was lower than 20%.

#### Statistical methods and data synthesis

Network meta-analysis was performed using odds ratio (OR) for the incidence of oral mucositis with a 95% confidence interval (CI) for the indirect and mixed comparisons. We checked for similarity, transitivity, and consistency. Transitivity was judged clinically, whereas consistency was judged formally [18]. We tested for possible global and local inconsistency by performing a χ2 test and by side-splitting, respectively. We estimated the ranking probabilities of being at each possible rank for each intervention. Comparison-adjusted funnel plots were employed to assess publication bias. In addition, sensitivity analysis was performed to determine the effect of each study by excluding a study with a high risk of bias or studies which could cause global or local inconsistency. Statistical evaluation of inconsistency and the production of network graphs and figures were performed using the network and network graphs packages in STATA version 15 (STATA Corporation, College Station, TX, USA). The Begg’s and Egger’s tests were used to detect publication bias.

## Results

### Search results

**Fig 1:**
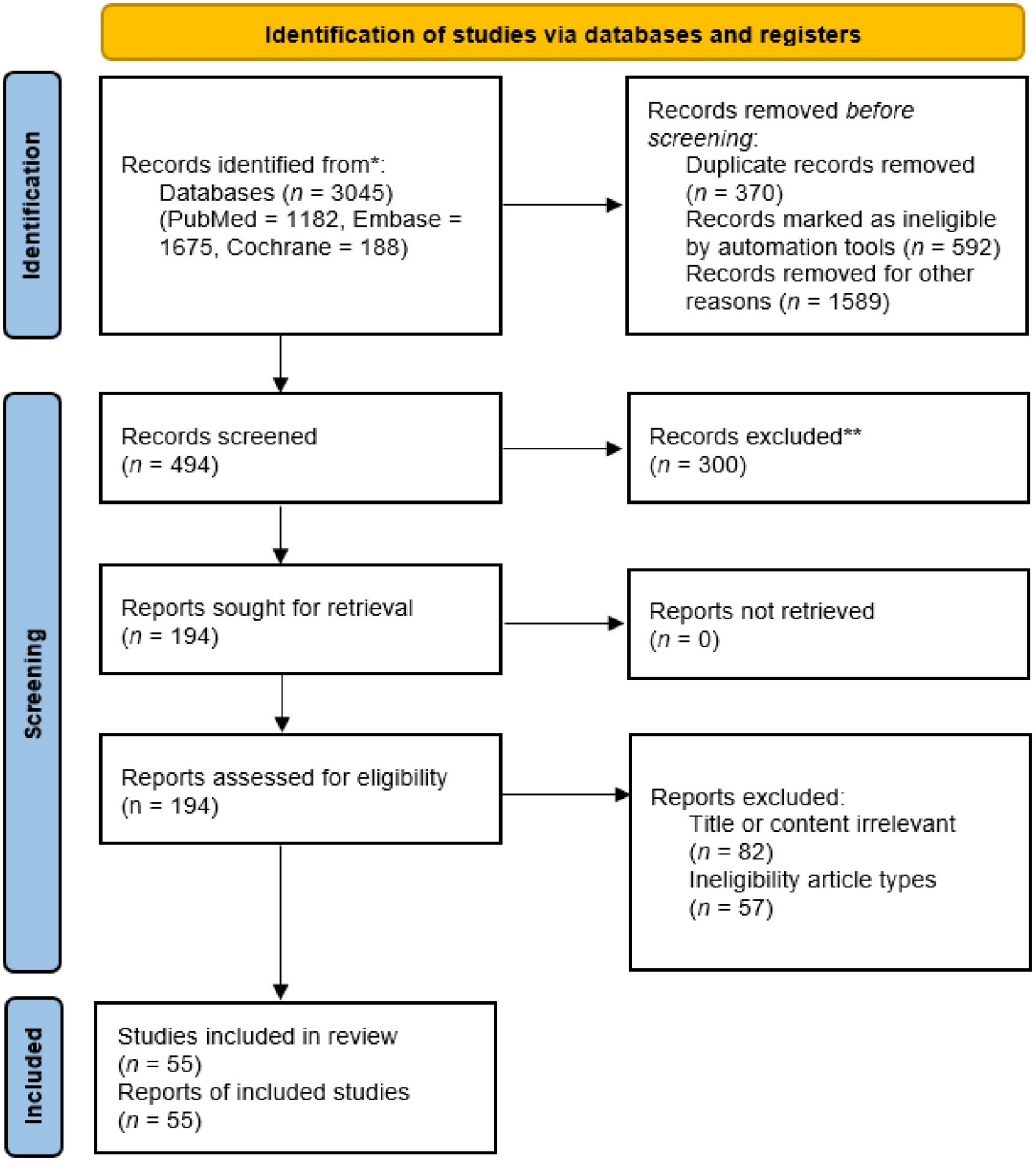
Flow diagram of the studies identified. *Consider, if feasible to do so, reporting the number of records identified from each database or register searched (rather than the total number across all databases/registers). **If automation tools were used, indicate how many records were excluded by a human and how many were excluded by automation tools.

We identified 3,045 records from PubMed, EMBASE, and Cochrane electronic databases. Three hundred seventy studies were removed due to duplication, 592 studies were removed due to non-RCTs, and 1,589 studies were removed due to not being the targets in this study. After the exclusion of these studies, we reviewed 194 studies based on title and abstract, and 139 studies were removed because of irrelevant records. Finally, 55 studies matched our inclusion criteria. The Systematic Reviews and Meta-Analyses (PRISMA) flowchart shows the detailed process of study selection (Fig 1).

### Eligible studies and patient characteristics

The basic characteristics of the eligible studies are presented in the Table 1. All included studies were published in English and randomized control trials, between 1994 and 2019. Most of the included studies have two arms, and only 2 studies have three arms. The risk of bias assessment of the 55 included trials is summarized in S1 Fig. The included studies encompassed 3,552 participants mostly with head and neck cancer.

**Table 1:** Characteristics of included studies.

Study	Study design	Cancer type	Chemoradiation (C)/Radiotherapy (R)	Number	Intervention	Events/Number (Grade 3 or 4 OM)
Lopez-Vaquero (2017) [19]	RCT	Head and neck	CR	25 24	Glutamine Placebo	1/25 2/24
Tanaka (2015) [20]	RCT	Esophageal	C	10 10	Glutamine Placebo	3/10 2/10
Tsujimoto (2015) [21]	RCT	Head and neck	C	20 20	Glutamine Placebo	18/20 20/20
Huang (2000) [22]	Randomized trial	Head and neck	R	8 9	Glutamine Placebo	0/8 4/9
Cerchietti (2006) [23]	RCT	Head and neck	C	14 15	Glutamine Placebo	0/14 5/15
Choi (2007) [24]	RCT	Advanced solid tumors	C	22 29	Glutamine Placebo	1/22 6/29
Peterson (2007) [25]	RCT	Breast	C	163 163	Glutamine Placebo	2/163 11/163
Okuno (1999) [26]	RCT	Undefined	C	66 68	Glutamine Placebo	4/66 5/68
Coghlin Dickson (2000) [27]	RCT	Hematologic (HSCT)	R	29 29	Glutamine Placebo	19/29 18/29
Jebb (1994) [28]	RCT	Advanced GI cancers	C	17 17	Glutamine Placebo	5/17 4/17
Skubitz (1996) [29]	RCT	Various	C	14 14	Glutamine Placebo	0/14 1/14
Nihei (2018) [30]	RCT	Colorectal or breast	C	34 33	Glutamine Placebo	11/34 19/33
Pathak (2019) [31]	RCT	Oropharynx and Larynx Carcinoma	CR	30 30	Glutamine Placebo	12/30 27/30
Huang (2019) [32]	RCT	Head and neck	R	31 33	Glutamine Placebo	17/31 26/33
Diwan (2018) [33]	RCT	Head and neck	R	30 30	Glutamine Placebo	4/30 7/30
Pattanayak (2016) [34]	RCT	Head and neck	CR	81 81	Glutamine Placebo	0/81 61/81
Amanat et al. (2017) [35]	RCT	Head and neck	R	41 41	Honey Placebo	2/41 7/41
Rao et al. (2017) [36]	RCT	Head and neck	R	25 25	Honey Povidone-iodine	8/25 12/24
Jayalekshmi et al. (2016) [37]	RCT	Head and neck	R	14 14	Honey Placebo	1/14 9/14
Eslami et al. (2016) [38]	Randomized trial	Acute lymphoblastic leukemia	C	24 24	Chlorhexidine Honey	9/24 1/24
Sahebjamee et al. (2015) [39]	RCT	Head and neck	R	13 13	Aloe Benzydamine	5/13 4/13
Hawley et al. (2014) [40]	RCT	Head and neck	R	40 41	Honey Placebo	14/40 18/41
Rao et al. (2014) [41]	RCT	Head and neck	R	39 40	Curcumin Povidone-iodine	14/39 34/40
Jayachandran and Balaji (2012) [42]	RCT	Head and neck	R	20 20 20	Honey Benzydamine Placebo	2/20 10/20 16/20
Roopashri et al. (2011) [43]	RCT	Head and neck	R	25 25 25	Povidone-iodine Chlorhexidine Placebo	2/25 3/25 4/25
Panahi et al. (2010) [44]	RCT	malignant disorders	C	15 15	Allopurinol Placebo	13/15 15/15
Khanal et al. (2010) [45]	RCT	oral carcinoma	R	20 20	Honey Lignocaine	1/20 15/20
Sorensen et al. (2008) [46]	RCT	Gastrointestinal Malignancies	C	70 64	Chlorhexidine Placebo	20/70 31/64
Cheng et al. (2006) [47]	RCT	Head and neck	R	7 7	Chlorhexidine Benzydamine	3/7 2/7
Vokurka et al. (2005) [48]	RCT	Autologous transplantation	C	37 65	Povidone-iodine Placebo	32/37 29/65
Dazzi et al. (2003) [49]	RCT	Autologous transplantation	C	46 44	GM-CSF Placebo	15/46 17/44
Costa et al. (2003) [50]	RCT	Acute lymphoblastic leukemia	C	7 7	Chlorhexidine Placebo	1/7 5/7
Nottage et al. (2003) [51]	RCT	Gastrointestinal Malignancies	C	41 39	Sucralfate Placebo	3/41 0/39
Castagna et al. (2001) [52]	RCT	bone marrow transplantation	C	51 51	Sucralfate Placebo	15/51 24/51
Cengiz et al. (1999) [53]	RCT	Head and neck	R	18 10	Sucralfate Placebo	9/18 9/10
Adamietz et al. (1998) [54]	RCT	Head and neck	CR	20 20	Povidone-iodine Placebo	4/20 13/20
Foote et al. (1994) [55]	RCT	Head and neck	R	25 27	Chlorhexidine Placebo	22/25 21/27
Alvi et al. (2013) [56]	RCT	Head and neck	R	30 30	Honey Placebo	4/30 12/30
Biswal et al. (2003) [57]	RCT	Nasopharynx, larynx	R	20 20	Honey Placebo	0/20 5/20
Rashad et al. (2010) [58]	RCT	Head and neck	R	20 20	Honey Placebo	0/20 7/20
Bardy et al. (2012) [59]	RCT	Head and neck	C	64 63	Honey Placebo	51/64 47/63
Charalambous et al. (2018) [60]	RCT	Head and neck	C	36 36	Honey Placebo	1/36 19/36
Abbasi et al. (2007) [61]	RCT	Head and neck	CR	14 10	Allopurinol Placebo	5/14 10/10
Pitten et al. (2003) [62]	RCT	leukopenia	C	24 23	Chlorhexidine Placebo	9/24 2/23
Schneider et al. (1999) [63]	RCT	Head and neck	CR	8 6	G-CSF Placebo	1/8 3/6
Su et al. (2006) [64]	RCT	Head and neck	R	19 21	G-CSF Placebo	4/19 11/21
Rahn et al. (1997) [65]	RCT	Head and neck	R	20 20	Povidone-iodine Placebo	9/20 20/20
Sharma et al. (2011) [66]	RCT	Head and neck	CR	93 95	Probiotics Placebo	49/93 73/95
Jiang et al. (2018) [67]	RCT	nasopharyngeal carcinoma	CR	58 35	Probiotics Placebo	9/58 16/35
De Sanctis et al. (2019) [68]	RCT	Head and neck	R	32 36	Probiotics Placebo	13/32 15/36
Mansourian et al. (2015) [69]	RCT	Head and neck	R	19 18	Curcumin Placebo	0/19 7/18
Delavarian et al. (2019) [70]	RCT	Head and neck	R	15 14	Curcumin Placebo	10/15 12/14
Arun et al. (2019) [71]	RCT	Head and neck	C	30 31	Curcumin Placebo	0/30 4/31
Su et al. (2004) [72]	RCT	Head and neck	R	28 30	Aloe Placebo	23/28 28/30
Puataweepong et al. (2009) [73]	RCT	Head and neck	R	30 31	Aloe Placebo	16/30 27/31

### Network geometry and testing for inconsistency

**Fig 2:**
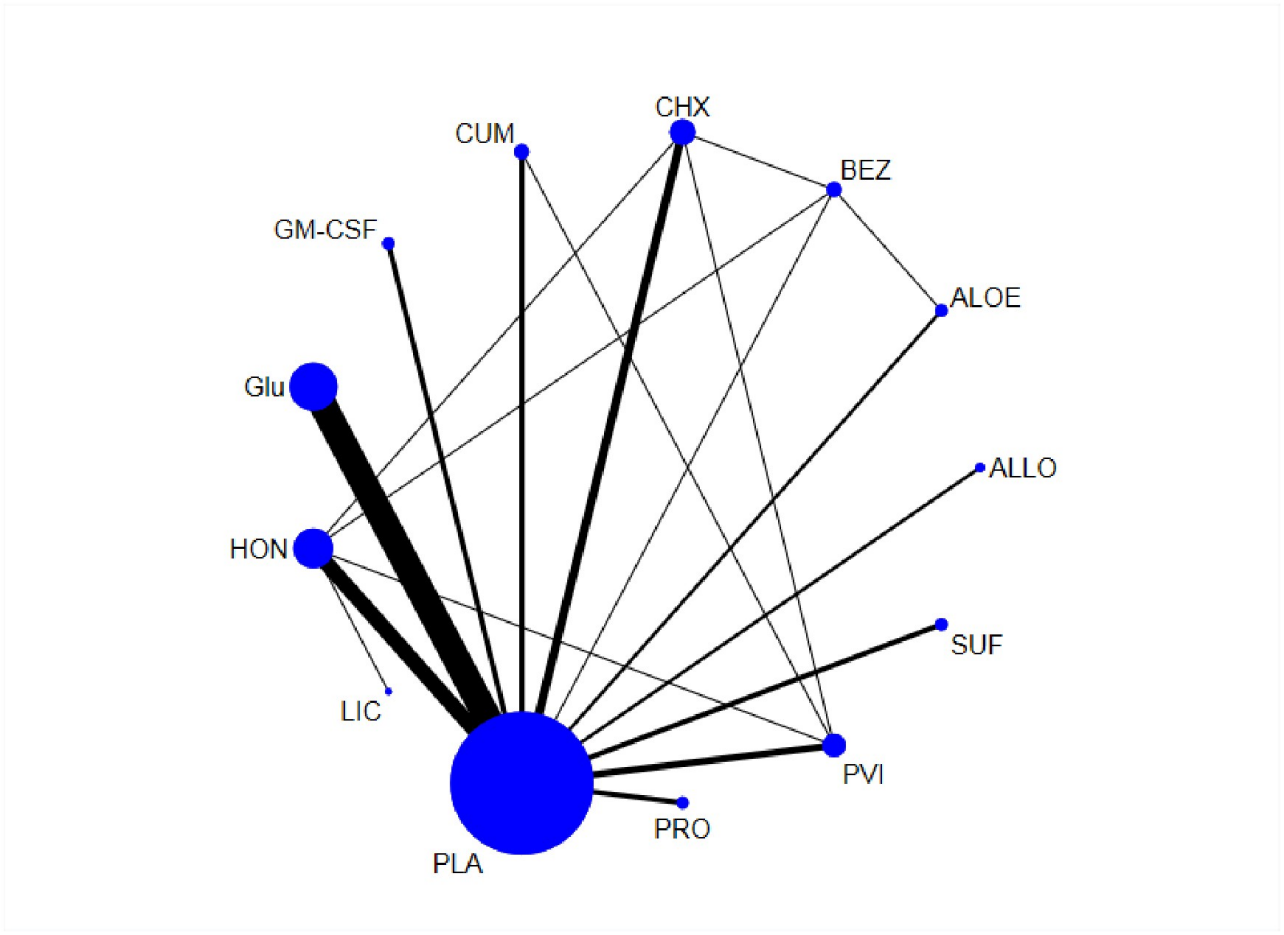
Evidence network of the RCTs in the network meta-analysis. Abbreviation: ALOE, aloe; ALLO, allopurinol; BEZ, benzydamine; CHX, chlorhexidine; CUM, Curcumin; Glu, glutamine; GM-CSF, granulocyte-macrophage colony-stimulating factor; HON, honey; LIC, lignocaine; PLA, placebo; PRO, probiotics; PVI, povidone-iodine; SUF, sucralfate.

The network constructions are presented in Fig 2. The p-value was higher than 0.05 (p = 0.9555) for the test of inconsistency at the overall level. No p-values were lower than 0.05 for the test of local inconsistency (S2 Fig). Significance was not found in any of the global or local tests, indicating that the consistency assumption was accepted.

### Treatment effect of moderate to severe oral mucositis

**Fig 3:**
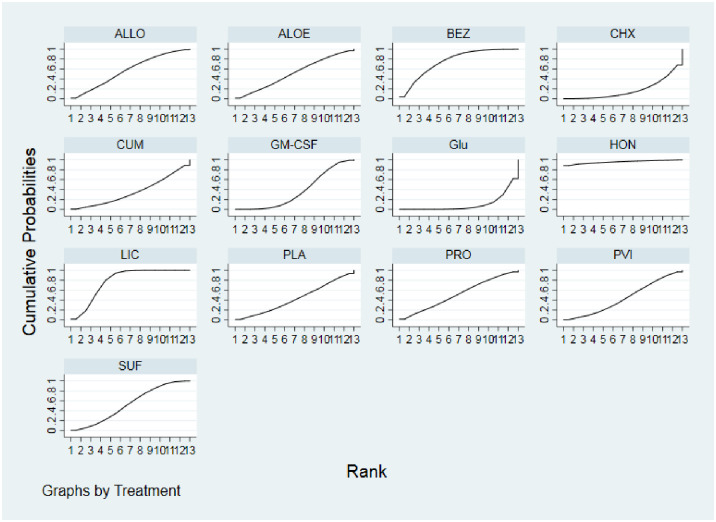
Rankograms for the network shows the probability of the incidence of moderate-severe oral mucositis of each treatment in patients with cancer. Abbreviation: ALOE, aloe; ALLO, allopurinol; BEZ, benzydamine; CHX, chlorhexidine; CUM, Curcumin; Glu, glutamine; GM-CSF, granulocyte-macrophage colony-stimulating factor; HON, honey; LIC, lignocaine; PLA, placebo; PRO, probiotics; PVI, povidone-iodine; SUF, sucralfate.

Network meta-analysis showed that, in comparison with placebo, honey ranked the best for the incidence of moderate-severe oral mucositis prevention and treatment (OR: 0.01, 95%CI 0.00 to 0.45), followed by lignocaine (OR: 0.07, 95%CI 0.00 to 0.95), benzydamine (OR: 0.07, 95%CI 0.00 to 1.19), allopurinol (OR: 0.22, 95%CI 0.01 to 4.84), sucralfate (OR: 0.12, 95%CI 0.01 to 2.10), aloe (OR: 0.17, 95%CI 0.01 to 3.67), probiotics (OR: 0.13, 95%CI 0.01 to 2.80), povidone-iodine (OR: 0.16, 95%CI 0.01 to 3.02), all of which ranked higher than placebo (Fig 3, Tables 2 and 3). However, granulocyte-macrophage colony-stimulating factor (GM-CSF), curcumin chlorhexidine, and glutamine were ranked lower than placebo. However, this network meta-analysis suggested that honey with the highest probability of preventing moderate-severe OM induced by chemo/radiotherapy in patients with all cancers. The surface under cumulative ranking curve (SUCRA) values for honey were 0.95, followed by lignocaine (SUCRA, 0.81) and benzydamine (SUCRA, 0.78).

**Table 2:** Rank probability to be the best treatment (PrBest) by the moderate-severe oral mucositis of each treatment of patients with cancer.

Treatment	Pbest
Honey	85.6%
Benzydamine	5.1%
Probiotics	2.7%
Lignocaine	2.1%
Sucralfate	1.2%
Aloe	1.1%
Curcumin	0.09%
Povidone-iodine	0.07%
Allopurinol	0.04%
Placebo	0.02%
GM-CSF	0%
Glutamine	0%
Chlorhexidine	0%

**Table 3:** Results of the incidence of moderate-severe oral mucositis; results presented as constant odds ratios between all competing interventions with 95% confidence intervals. *Comparisons of treatments should be read from left to right. The rate ratio lower than 1 favors the top left treatment. The treatments have been sorted from left to right according to treatment ranking. Statistically significant differences between regimens are shown in bold with green background.

**HON**												
0.11 (0.00,2.60)	**LIC**											
0.10 (0.00,2.90)	0.98 (0.34,2.78)	**BEZ**										
0.03 (0.00,1.14)	0.31 (0.06,1.51)	0.31 (0.05,2.00)	**ALLO**									
0.06 (0.00,1.68)	0.57 (0.19,1.72)	0.58 (0.14,2.44)	1.85 (0.27,12.78)	**SUF**								
0.04 (0.00,1.47)	0.41 (0.08,2.03)	0.42 (0.07,2.39)	1.33 (0.21,8.55)	0.72 (0.11,4.90)	**ALOE**							
0.06 (0.00,2.09)	0.54 (0.11,2.77)	0.55 (0.08,3.86)	1.76 (0.18,17.32)	0.95 (0.13,6.94)	1.33 (0.13,13.20)	**PRO**						
0.05 (0.00,1.44)	0.43 (0.11,1.60)	0.44 (0.08,2.35)	1.40 (0.18,11.06)	0.76 (0.14,4.24)	1.06 (0.13,8.44)	0.79 (0.10,6.48)	**PVI**					
**0.01 (0.00,0.45)**	**0.07 (0.00,0.95)**	0.07 (0.00,1.19)	0.22 (0.01,4.84)	0.12 (0.01,2.10)	0.17 (0.01,3.67)	0.13 (0.01,2.80)	0.16 (0.01,3.02)	**PLA**				
**0.03 (0.00,0.83)**	**0.30 (0.15,0.61)**	0.31 (0.09,1.08)	0.97 (0.17,5.59)	0.53 (0.14,1.96)	0.73 (0.13,4.26)	0.55 (0.09,3.30)	0.70 (0.16,3.11)	4.39 (0.29,67.34)	**GM-CSF**			
0.04 (0.00,1.32)	0.36 (0.08,1.65)	0.37 (0.06,2.34)	1.18 (0.13,10.69)	0.64 (0.10,4.18)	0.89 (0.10,8.14)	0.67 (0.07,6.27)	0.85 (0.11,6.30)	5.33 (0.25,111.69)	1.22 (0.23,6.48)	**CUM**		
**0.01 (0.00,0.35)**	**0.10 (0.02,0.45)**	**0.10 (0.02,0.61)**	0.31 (0.03,2.86)	**0.17 (0.03,0.86)**	0.24 (0.03,2.16)	0.18 (0.02,1.68)	0.22 (0.03,1.69)	1.41 (0.07,29.84)	0.32 (0.06,1.75)	0.26 (0.03,2.29)	**CHX**	
**0.02 (0.00,0.38)**	**0.17 (0.07,0.39)**	**0.17 (0.05,0.60)**	0.54 (0.09,3.20)	0.29 (0.08,1.04)	0.41 (0.07,2.27)	0.31 (0.05,1.96)	0.39 (0.08,1.86)	2.44 (0.15,39.02)	0.56 (0.19,1.67)	0.46 (0.08,2.59)	1.74 (0.31,9.73)	**Glu**

Abbreviation: ALOE, aloe; ALLO, allopurinol; BEZ, benzydamine; CHX, chlorhexidine; CUM, Curcumin; Glu, glutamine; GM-CSF, granulocyte-macrophage colony-stimulating factor; HON, honey; LIC, lignocaine; PLA, placebo; PRO, probiotics; PVI, povidone-iodine; SUF, sucralfate.

### Subgroup by head and neck cancer

The network meta-analysis for the incidence of moderate-severe oral mucositis of each treatment in patients with head and neck cancer was based on 39 trials. Results from network meta-analysis that honey is the best intervention to prevent or treat moderate-severe grade oral mucositis than placebo (OR: 0.00, 95%CI 0.00 to 0.36) with the highest probability of ranking the best (85.5%; S3 Fig, S1 and S3 Tables). However, the honey with the highest probability of preventing moderate-severe OM induced by chemo/radiotherapy in patients with head and neck cancer (SUCRA, 0.96), followed by lignocaine (SUCRA, 0.83), benzydamine (SUCRA, 0.79), and povidone-iodine. (SUCRA, 0.59).

### Subgroup by radiotherapy

The network meta-analysis for radiotherapy-induced moderate-severe oral mucositis of each treatment in patients with cancer was based on 26 trials. Results from network meta-analysis that honey is the best intervention for preventing or treatment of moderate-severe grade oral mucositis than placebo (OR: 0.03, 95%CI 0.00 to 0.67) with the highest probability of ranking the best (85.9%; S4 Fig, S2 and S4 Tables). However, the honey with the highest probability of preventing moderate-severe OM induced by radiotherapy in patients with cancers (SUCRA, 0.97), followed by lignocaine (SUCRA, 0.79), benzydamine (SUCRA, 0.77), and GM-CSF (SUCRA, 0.54).

### Adverse events

Most of the included studies did not describe the occurrence of adverse events to therapy with these agents. Few studies reported the adverse events of the interventions.

#### Glutamine

Ten studies did not assess the safety issues [20–22, 24, 26–29, 32, 33]. Six studies found that patients in the glutamine group experienced no side effects or significant differences between the glutamine group and the control group [19, 23, 25, 30, 31, 34].

#### Honey

All of the studies did not examine the safety of honey [35–37, 40, 42, 56–60]. Two studies compared to honey and lignocaine or chlorhexidine and also did not evaluate the safety outcome [38, 45].

#### Aloe

All of the studies did not examine the safety of aloe [39, 72, 73].

#### Curcumin

Three studies examined the safety of curcumin. Two studies found that patients in the curcumin group experienced no side effects or discomfort caused by curcumin [70, 71]. In one study, two patients experienced nausea after the administration of curcumin gel [69]. One study does not assess the safety issue [41].

#### Probiotics

Only one study examined the safety of probiotics. The study found that patients in the probiotics group experienced no side effects caused by probiotics [66]. Two studies did not assess the safety issues [67, 68].

### Publication bias, and sensitivity analysis

**Fig 4:**
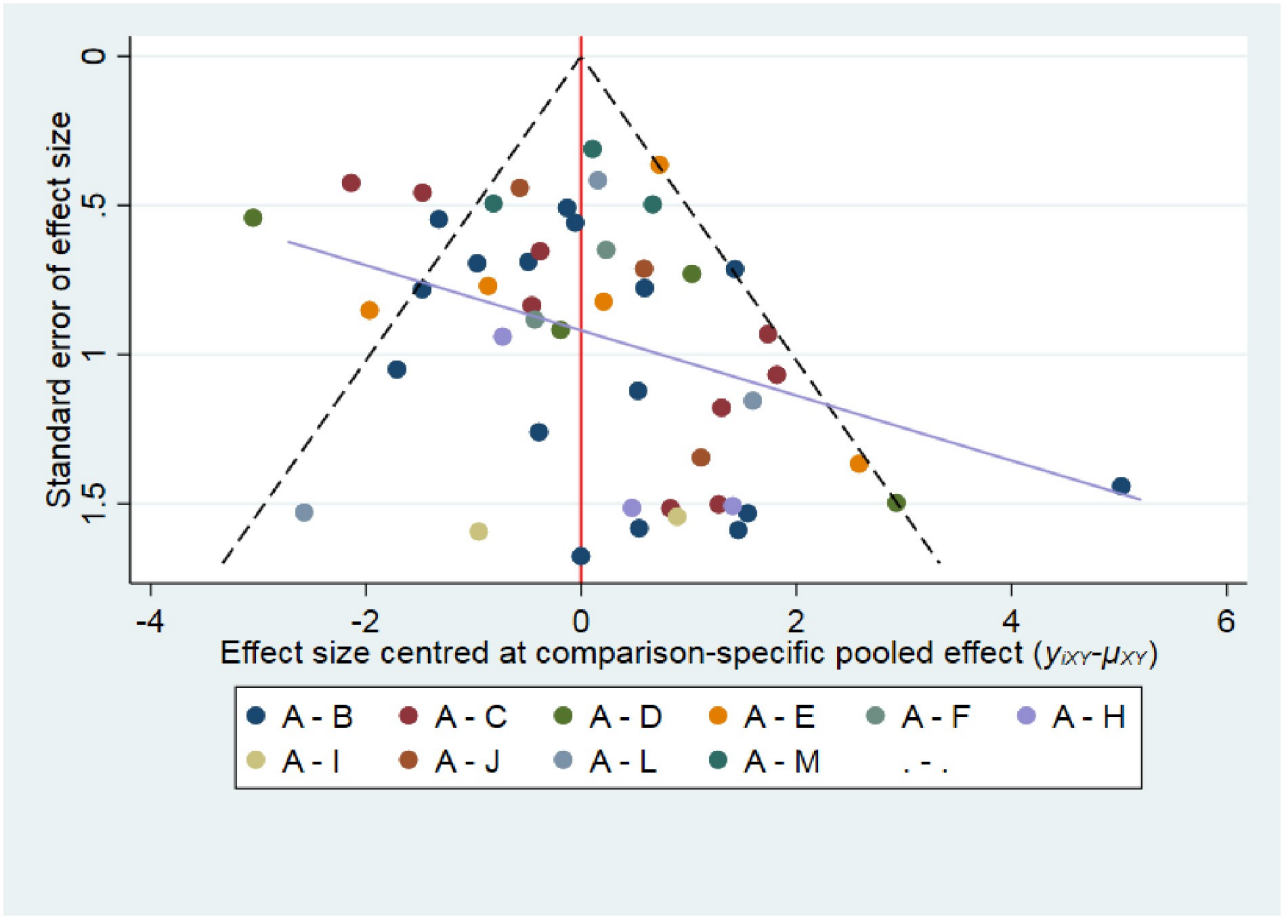
Comparison-adjusted funnel plot for the selected studies with the incidence of moderate-severe oral mucositis. Abbreviation: A, placebo; B, glutamine; C, honey; D, povidone-iodine; E, chlorhexidine; F, aloe; G, benzydamine; H, Curcumin; I, allopurinol; J, granulocyte-macrophage colony-stimulating factor; K, lignocaine; L, sucralfate; M, probiotics. Note: Comparisons including only one study (when present) have been excluded.

The comparison-adjusted funnel plots did not reveal any evidence of apparent asymmetry (Fig 4). No significant publication bias was observed. We also do sensitivity analysis. We excluded two studies with a high risk of bias one by one [43, 50], which resulted in similar results of the incidence of moderate-severe oral mucositis in comparison with our basic analysis (S5 Table).

## Discussion

Several interventions are effective in preventing and treating OM. However, current evidence is based on a direct meta-analysis. Network meta-analysis is a technique for comparing multiple treatments simultaneously in a single analysis by combining direct and indirect results. This network meta-analysis investigated available evidence on the efficacy of preventing the risk of chemotherapy- or radiation therapy-induced moderate-severe oral mucositis of various interventions for patients with cancer. The results of this network meta-analysis showed that honey and lignocaine were more effective than placebo.

A previous network meta-analysis conducted by Yu et al. This network meta-analysis was compared nine oral care solutions (allopurinol, aloe, benzydamine, chlorhexidine, curcumin, granulocyte-macrophage colony-stimulating factor, honey, povidone-iodine, and sucralfate), including 28 RCTs with 1,861 patients. The results of network meta-analysis showed that chlorhexidine, benzydamine, honey, and curcumin were more effective than placebo (p < 0.05) [74]. Another meta-analysis demonstrated that honey significantly reduced the severity of grade 3 and 4 OM [75]. This result was similar to our study [76]. Some network meta-analyses are comparing the prevention or treatment of OM in cancer patients, but the research directions are slightly different. There is a study of OM in patients with head and neck cancer who received radiotherapy, and in addition to standard oral care, they also added low-level laser [77]. However, our study only analyzed methods that are easily accessible to patients and do not require special interventions such as low-level laser or cryotherapy [77, 78].

Honey has been proven to have anti-inflammatory, antioxidant, antimicrobial, and rapid tissue-healing properties [79, 80]. The mechanism of honey that can prevent OM is attributed to its antimicrobial property. This property with high osmolality is sufficient to inhibit microbial growth and its production of hydrogen peroxide [81]. Honey has been demonstrated to improve the epithelization of tissue when used for wound dressing to improve wound healing. Benzydamine mouthwash, an anti-inflammatory agent, significantly reduces erythema and ulceration. Lignocaine application is an anesthetic agent but has no anti-inflammatory properties. These agents may reduce erythema, ulceration, and pain of the OM.

Almost all patients with head and neck cancer who receive radiation therapy occur in OM [82]. We have also conducted subgroups by chemotherapy and radiation therapy-induced OM in head and neck cancer patients, and radiation therapy-induced OM in cancer patients. Honey still has the highest probability of preventing moderate-severe OM in the subgroup analysis.

Among the adverse events of treatment, few studies reported adverse events of interventions. In some sporadic reports of curcumin used in OM, only one study reported nausea in two patients. Glutamine or probiotics had no side effects or significant differences between the glutamine or probiotics group and the control group. While this study was the most effective honey, none of the studies examined the safety of honey.

This study has several limitations. First, this study was not assessing the side effects of different interventions. Because these data on side effects in different interventions were not available. Second, some of the treatments, including lignocaine and allopurinol, were covered in 1 and 2 studies with a small number of patients. Third, regarding the quality of evidence (GRADE), several comparisons were assessed with low quality which may restrict the interpretation of these results.

## Conclusions

This network meta-analysis results indicate that honey and lignocaine may be the preferred choices for patients with cancers to prevent or treat OM. Further large randomized controlled trials providing a higher level of evidence should be conducted to confirm our findings.

## Supporting information

S1 Fig The risk of bias summary. (TIF)

S2 Fig Side-splitting results of the selected studies with the incidence of moderate-severe oral mucositis for evaluating local inconsistency. (**Abbreviation**: A, placebo; B, glutamine; C, honey; D, povidone-iodine; E, chlorhexidine; F, aloe; G, benzydamine; H, Curcumin; I, allopurinol; J, granulocyte-macrophage colony-stimulating factor; K, lignocaine; L, sucralfate; M, probiotics.).
(TIF)

S3 Fig Rankograms for the network showing the probability for the incidence of moderate-severe oral mucositis of each treatment in patients with head and neck cancer. (**Abbreviation**: ALOE, aloe; ALLO, allopurinol; BEZ, benzydamine; CHX, chlorhexidine; CUM, Curcumin; Glu, glutamine; GM-CSF, granulocyte-macrophage colony-stimulating factor; HON, honey; LIC, lignocaine; PLA, placebo; PRO, probiotics; PVI, povidone-iodine; SUF, sucralfate.).
(TIF)

S4 Fig Rankograms for the network showing the probability for the incidence of radiotherapy-induced moderate-severe oral mucositis of each treatment in patients with cancer. (**Abbreviation**: ALOE, aloe; ALLO, allopurinol; BEZ, benzydamine; CHX, chlorhexidine; CUM, Curcumin; Glu, glutamine; GM-CSF, granulocyte-macrophage colony-stimulating factor; HON, honey; LIC, lignocaine; PLA, placebo; PRO, probiotics; PVI, povidone-iodine; SUF, sucralfate.).
(TIF)

S1 Checklist PRISMA 2009 checklist. (DOC)

S1 Table Rank probability to be the best treatment (PrBest) by the incidence of moderate-severe oral mucositis of each treatment in patients with head and neck cancer. (DOCX)

S2 Table Rank probability to be the best treatment (PrBest) by the incidence of radiotherapy-induced moderate-severe oral mucositis of each treatment in patients with cancer. (DOCX)

S3 Table Results of the incidence of moderate-severe oral mucositis in patients with head and neck cancer. (DOCX)

S4 Table Results of the incidence of radiotherapy-induced moderate-severe oral mucositis in patients with cancer. (DOCX)

S5 Table Results of the incidence of moderate-severe oral mucositis. a. Excluding the study performed by Roopashri et al. b. Excluding the study performed by Roopashri and Costa et al.
(DOCX)

## References

[1] Miao J, Liu X, Wu C, Kong H, Xie W, Liu K. Effects of acupressure on chemotherapy-induced nausea and vomiting-a systematic review with meta-analyses and trial sequential analysis of randomized controlled trials. Int J Nurs Stud 2017;70:27–37. doi: 10.1016/j.ijnurstu.2017.02.014 . 28231440

[2] Raber-Durlacher JE, Weijl NI, Abu Saris M, de Koning B, Zwinderman AH, Osanto S. Oral mucositis in patients treated with chemotherapy for solid tumors: a retrospective analysis of 150 cases. Support Care Cancer. 2000;8(5):366–71. doi: 10.1007/s005200050004 . 10975685

[3] Maria OM, Eliopoulos N, Muanza T. Radiation-induced oral mucositis. Front Oncol 2017;7:89. doi: 10.3389/fonc.2017.00089 . 28589080 PMC5439125

[4] Oronsky B, Goyal S, Kim MM, Cabrales P, Lybeck M, Caroen S, et al. A review of clinical radioprotection and chemoprotection for oral mucositis. Transl Oncol 2018;11:771–8. doi: 10.1016/j.tranon.2018.03.014 . 29698934 PMC5918142

[5] Niikura N, Ota Y, Hayashi N, Naito M, Kashiwabara K, Watanabe K, et al. Evaluation of oral care to prevent oral mucositis in estrogen receptor-positive metastatic breast cancer patients treated with everolimus (Oral Care-BC): randomized controlled phase III trial, Jpn. J Clin Oncol 2016;46:879–82. doi: 10.1093/jjco/hyw077 . 27365521

[6] Wasko-Grabowska A, Rzepecki P, Oborska S, Barzal J, Mlot B, Gawronski K, et al. A supersaturated calcium phosphate solution seems to effectively prevent and treat oral mucositis in haematopoietic stem cell transplanted cancer patients—single centre experience. J BUON 2012;17:363–8. . 22740219

[7] Garavito AA, Cardona AF, Reveiz L, Ospina E, Yepes A, Ospina V. Colchicine mouth washings to improve oral mucositis in patients with hematological malignancies: a clinical trial. Palliat Support Care 2008;6:371–6. doi: 10.1017/S147895150800059X . 19006592

[8] Peng TR, Lin HH, Yang LJ, Wu TW. Effectiveness of glutamine in the management of oral mucositis in cancer patients: a meta-analysis of randomized controlled trials. Support Care Cancer. 2021;29(8):4885–4892. doi: 10.1007/s00520-021-06060-9 . 33598734

[9] An W, Li S, Qin L. Role of honey in preventing radiation-induced oral mucositis: a meta-analysis of randomized controlled trials. Food Funct. 2021;12(8):3352–3365. doi: 10.1039/d0fo02808h . 33900311

[10] Zhang L, Tang G, Wei Z. Prophylactic and Therapeutic Effects of Curcumin on Treatment-Induced Oral Mucositis in Patients with Head and Neck Cancer: A Meta-Analysis of Randomized Controlled Trials. Nutr Cancer 2021;73(5):740–749. doi: 10.1080/01635581.2020.1776884 . 32515617

[11] Lima ICGDS, de Fátima Souto Maior L, Gueiros LAM, Leão JC, Higino JS, Carvalho AAT. Clinical applicability of natural products for prevention and treatment of oral mucositis: a systematic review and meta-analysis. Clin Oral Investig 2021;25(6):4115–4124. doi: 10.1007/s00784-020-03743-1 . 33409696

[12] Lumley T. Network meta-analysis for indirect treatment comparisons. Stat Med 2002;21:2313–24. doi: 10.1002/sim.1201 . 12210616

[13] Hutton B, Salanti G, Caldwell DM, Chaimani A, Schmid CH, Cameron C, et al. The PRISMA extension statement for reporting of systematic reviews incorporating network meta-analyses of health care interventions: checklist and explanations. Ann Intern Med 2015;162(11):777–84. doi: 10.7326/M14-2385 . 26030634

[14] Higgins JP, Altman DG, Gøtzsche PC, Jüni P, Moher D, Oxman AD, et al. Cochrane Bias Methods Group; Cochrane Statistical Methods Group. The Cochrane Collaboration’s tool for assessing the risk of bias in randomised trials. BMJ 2011;343:d5928. doi: 10.1136/bmj.d5928 . 22008217 PMC3196245

[15] Cox JD, Stetz J, Pajak TF. Toxicity criteria of the Radiation Therapy Oncology Group (RTOG) and the European Organization for Research and Treatment of Cancer (EORTC). Int J Radiat Oncol Biol Phys 1995;31(5):1341–6. doi: 10.1016/0360-3016(95)00060-C . 7713792

[16] World Health Organization. Handbook for Reporting Results of Cancer Treatment. Geneva: World Health Organization; 1979. WHO offset publication no. 48.

[17] National Cancer Institute. Common Terminology Criteria for Adverse Events (CTCAE), 2017, version 5.0. European Organization for Research and Treatment of Cancer.

[18] Rouse B, Chaimani A, Li T. Network meta-analysis: an introduction for clinicians. Intern Emerg Med 2017;12(1):103–111. doi: 10.1007/s11739-016-1583-7 . 27913917 PMC5247317

[19] Lopez-Vaquero D, Gutierrez-Bayard L, Rodriguez-Ruiz JA, Saldaña-Valderas M, Infante-Cossio P. Double-blind randomized study of oral glutamine on the management of radio/ chemotherapy-induced mucositis and dermatitis in head and neck cancer. Mol Clin Oncol 2017;6(6):931–936. doi: 10.3892/mco.2017.1238 . 28588793 PMC5451869

[20] Tanaka Y, Takahashi T, Yamaguchi K, Osada S, Shimokawa T, Yoshida K. Elemental diet plus glutamine for the prevention of mucositis in esophageal cancer patients receiving chemotherapy: a feasibility study. Support Care Cancer 2016; 24(2):933–941. doi: 10.1007/s00520-015-2864-9 . 26266659 PMC4689762

[21] Tsujimoto T, Yamamoto Y, Wasa M, Takenaka Y, Nakahara S, Takagi T, et al. L-glutamine decreases the severity of mucositis induced by chemoradiotherapy in patients with locally advanced head and neck cancer: a doubleblind, randomized, placebo- controlled trial. Oncol Rep 2015:33(1):33–39. doi: 10.3892/or.2014.3564 . 25351453 PMC4254677

[22] Huang EY, Leung SW, Wang CJ, Chen HC, Sun LM, Fang FM, et al. Oral glutamine to alleviate radiation-induced oral mucositis: a pilot randomized trial. Int J Radiat Oncol Biol Phys 2000;46(3):535–9. doi: 10.1016/s0360-3016(99)00402-2 . 10701731

[23] Cerchietti LC, Navigante AH, Lutteral MA, Castro MA, Kirchuk R, Bonomi M, et al. Double blinded, placebo-controlled trial on intravenous L-alanyl-Lglutamine in the incidence of oral mucositis following chemoradiotherapyin patients with head-and-neck cancer. Int J Radiat Oncol Biol Phys 2006;65(5):1330–1337. doi: 10.1016/j.ijrobp.2006.03.042 . 16765532

[24] Choi K, Lee SS, Oh SJ, Lim SY, Lim SY, Jeon WK, et al. The effect of oral glutamine on 5-fluorouracil/leucovorin-induced mucositis/stomatitis assessed by intestinal permeability test. Clin Nutr 2007;26(1):57–62. doi: 10.1016/j.clnu.2006.07.003 . 16949180

[25] Peterson DE, Jones JB, Petit RG. Randomized, placebo controlled trial of Saforis for prevention and treatment of oral mucositis in breast cancer patients receiving anthracycline-based chemotherapy. Cancer 2007;109(2):322–31. doi: 10.1002/cncr.22384 . 17154160

[26] Okuno SH, Woodhouse CO, Loprinzi CL, Sloan JA, LaVasseur BI, Clemens-Schutjer D, et al. Phase III controlled evaluation of glutamine for decreasing stomatitis in patients receiving fluorouracil (5-FU)-based chemotherapy. Am J Clin Oncol 1999;22(3):258–261. doi: 10.1097/00000421-199906000-00009 . 10362332

[27] Coghlin Dickson TM, Wong RM, Offrin RS, Shizuru JA, Johnston LJ, Hu WW, et al. Effect of oral glutamine supplementation during bone marrow transplantation. J Parenter Enter Nutr 2000;24(2):61–66. doi: 10.1177/014860710002400261 . 10772184

[28] Jebb SA, Osborne RJ, Maughan TS, Mohideen N, Mack P, Mort D, et al. 5-Fluorouracil and folimc acid-induced mucositis: no effect of oral glutamine supplementation. Br J Cancer 1994;70(4):732–735. doi: 10.1038/bjc.1994.385 . 7917930 PMC2033386

[29] Skubitz KM, Anderson PM. Oral glutamine to prevent chemotherapy induced stomatitis: a pilot study. J Lab Clin Med 1996;127(2):223–8. doi: 10.1016/s0022-2143(96)90082-7 . 8636652

[30] Nihei S, Sato J, Komatsu H, Ishida K, Kimura T, Tomita T, et al. The efficacy of sodium azulene sulfonate L-glutaminefor managing chemotherapy-induced oral mucositis in cancer patients: a prospective comparative study. J Pharm Health Care Sci 2018;4:20. doi: 10.1186/s40780-018-0114-2 . 30123519 PMC6088392

[31] Pathak S, Soni TP, Sharma LM, Patni N, Gupta AK. A randomized controlled trial to evaluate the role and efficacy of oralglutamine in the treatment of vhemo-radiotherapy-induced oral mucositis and dysphagia in patients with oropharynx and larynx carcinoma. Cureus 2019;11(6):e4855. doi: 10.7759/cureus.4855 . 31410338 PMC6684294

[32] Huang CJ, Huang MY, Fang PT, Chen F, Wang YT, Chen CH, et al. Randomized double blind, placebo- controlled trial evaluating oral glutamine on radiation-induced oral mucositis and dermatitis in head and neck cancer patients. Am J Clin Nutr 2019;109(3):606–614. doi: 10.1093/ajcn/nqy329 . 30753262 PMC6408208

[33] Diwan AK, Khan S. Assessing role of oral glutamine supplementation in radiation induced oral mucositis in head and neck cancers. Ann Int Med Dental Res 2018;4(2):1–6. doi: 10.21276/aimdr.2018.4.2.rt2

[34] Pattanayak L, Panda N, Dash MK, Mohanty S, Samantaray S. Management of chemoradiation-induced mucositis in head and neck cancers with oral glutamine. J Glob Oncol 2016;2(4):200–206. doi: 10.1200/JGO.2015.000786 . 28717702 PMC5497617

[35] Amanat A, Ahmed A, Kazmi A, Aziz B. The Effect of Honey on Radiation-induced Oral Mucositis in Head and Neck Cancer Patients. Indian J Palliat Care 2017;23(3):317–320. doi: 10.4103/IJPC.IJPC_146_16 . 28827938 PMC5545960

[36] Rao S, Hegde SK, Rao P, Dinkar C, Thilakchand KR, George T, et al. Honey Mitigates Radiation-Induced Oral Mucositis in Head and Neck Cancer Patients without Affecting the Tumor Response. Foods. 2017;6(9):77. doi: 10.3390/foods6090077 . 28878156 PMC5615289

[37] Jayalekshmi JL, Lakshmi R, Mukerji A. Honey on oral mucositis: A Randomized controlled trial. Gulf J Oncolog 2016;1(20):30–7. . 27050177

[38] Eslami H, Pouralibaba F, Falsafi P, Bohluli S, Najati B, Negahdari R, et al. Efficacy of Hypozalix spray and propolis mouthwash for prevention of chemotherapy-induced oral mucositis in leukemic patients: A double-blind randomized clinical trial. J Dent Res Dent Clin Dent Prospects. 2016;10(4):226–233. doi: 10.15171/joddd.2016.036 . 28096948 PMC5237669

[39] Sahebjamee M, Mansourian A, Hajimirzamohammad M, Zadeh MT, Bekhradi R, Kazemian A, et al. Comparative Efficacy of Aloe vera and Benzydamine Mouthwashes on Radiation-induced Oral Mucositis: A Triple-blind, Randomised, Controlled Clinical Trial. Oral Health Prev Dent 2015;13(4):309–15. doi: 10.3290/j.ohpd.a33091 . 25431805

[40] Hawley P, Hovan A, McGahan CE, Saunders D. A randomized placebo-controlled trial of manuka honey for radiation-induced oral mucositis. Support Care Cancer 2014;22(3):751–61. doi: 10.1007/s00520-013-2031-0 . 24221577

[41] Rao S, Dinkar C, Vaishnav LK, Rao P, Rao P, Rai MP, Fayad R, et al. The Indian Spice Turmeric Delays and Mitigates Radiation-Induced Oral Mucositis in Patients Undergoing Treatment for Head and Neck Cancer: An Investigational Study. Integr Cancer Ther 2014;13(3):201–10. doi: 10.1177/1534735413503549 . 24165896

[42] Jayachandran S, Balaji N. Evaluating the effectiveness of topical application of natural honey and benzydamine hydrochloride in the management of radiation mucositis. Indian J Palliat Care 2012;18(3):190–5. doi: 10.4103/0973-1075.105689 . 23439942 PMC3573473

[43] Roopashri G, Jayanthi K, Guruprasad R. Efficacy of benzydamine hydrochloride, chlorhexidine, and povidone iodine in the treatment of oral mucositis among patients undergoing radiotherapy in head and neck malignancies: A drug trail. Contemp Clin Dent 2011;2(1):8–12. doi: 10.4103/0976-237X.79292 . 22114446 PMC3220182

[44] Panahi Y, Ala S, Saeedi M, Okhovatian A, Bazzaz N, Naghizadeh MM. Allopurinol mouth rinse for prophylaxis of fluorouracil-induced mucositis. Eur J Cancer Care (Engl) 2010;19(3):308–12. doi: 10.1111/j.1365-2354.2008.01042.x . 19659665

[45] Khanal B, Baliga M, Uppal N. Effect of topical honey on limitation of radiation-induced oral mucositis: an intervention study. Int J Oral Maxillofac Surg 2010;39(12):1181–5. doi: 10.1016/j.ijom.2010.05.014 . 20832243

[46] Sorensen JB, Skovsgaard T, Bork E, Damstrup L, Ingeberg S. Double-blind, placebo-controlled, randomized study of chlorhexidine prophylaxis for 5-fluorouracil-based chemotherapy-induced oral mucositis with nonblinded randomized comparison to oral cooling (cryotherapy) in gastrointestinal malignancies. Cancer 2008;112(7):1600–6. doi: 10.1002/cncr.23328 . 18300265

[47] Kin-Fong Cheng K, Ka Tsui Yuen J. A pilot study of chlorhexidine and benzydamine oral rinses for the prevention and treatment of irradiation mucositis in patients with head and neck cancer. Cancer Nurs 2006;29(5):423–30. doi: 10.1097/00002820-200609000-00012 . 17006117

[48] Vokurka S, Bystrická E, Koza V, Scudlová J, Pavlicová V, Valentová D, et al. The comparative effects of povidone-iodine and normal saline mouthwashes on oral mucositis in patients after high-dose chemotherapy and APBSCT—results of a randomized multicentre study. Support Care Cancer 2005;13(7):554–8. doi: 10.1007/s00520-005-0792-9 . 15798915

[49] Dazzi C, Cariello A, Giovanis P, Monti M, Vertogen B, Leoni M, et al. Prophylaxis with GM-CSF mouthwashes does not reduce frequency and duration of severe oral mucositis in patients with solid tumors undergoing high-dose chemotherapy with autologous peripheral blood stem cell transplantation rescue: a double blind, randomized, placebo-controlled study. Ann Oncol 2003;14(4):559–63. doi: 10.1093/annonc/mdg177 . 12649101

[50] Costa EM, Fernandes MZ, Quinder LB, de Souza LB, Pinto LP. Evaluation of an oral preventive protocol in children with acute lymphoblastic leukemia. Pesqui Odontol Bras 2003;17:147–50. doi: 10.1590/s1517-74912003000200009 . 14569357

[51] Nottage M, McLachlan SA, Brittain MA, Oza A, Hedley D, Feld R, et al. Sucralfate mouthwash for prevention and treatment of 5-fluorouracil-induced mucositis: a randomized, placebo-controlled trial. Support Care Cancer 2003;11(1):41–7. doi: 10.1007/s00520-002-0378-8 . 12527953

[52] Castagna L, Benhamou E, Pedraza E, Luboinski M, Forni M, Brandes I, et al. Prevention of mucositis in bone marrow transplantation: a double blind randomised controlled trial of sucralfate. Ann Oncol 2001;12(7):953–5. doi: 10.1023/a:1011119721267 . 11521801

[53] Cengiz M, Ozyar E, Oztürk D, Akyol F, Atahan IL, Hayran M. Sucralfate in the prevention of radiation-induced oral mucositis. J Clin Gastroenterol 1999;28:40–3. doi: 10.1097/00004836-199901000-00009 . 9916664

[54] Adamietz IA, Rahn R, Böttcher HD, Schäfer V, Reimer K, Fleischer W. Prophylaxis with povidone-iodine against induction of oral mucositis by radiochemotherapy. Support Care Cancer 1998;6(4):373–7. doi: 10.1007/s005200050179 . 9695205

[55] Foote RL, Loprinzi CL, Frank AR, O’Fallon JR, Gulavita S, Tewfik HH, et al. Randomized trial of a chlorhexidine mouthwash for alleviation of radiation-induced mucositis. J Clin Oncol 1994;12:2630–3. doi: 10.1200/JCO.1994.12.12.2630 . 7989938

[56] Alvi Z, Mahmood A, Rasul S, Ali U, Arif S, Ishtiaq S, et al. Role of honey in prevention of radiation induced mucositis in head and neck cancer. Pakistan Armed Forces Med J 2013;63:379–383. https://pafmj.org/index.php/PAFMJ/article/view/2220

[57] Biswal BM, Zakaria A, Ahmad NM. Topical application of honey in the management of radiation mucositis: a preliminary study. Support Care Cancer 2003;11:242–248. doi: 10.1007/s00520-003-0443-y . 12673463

[58] Rashad UM, Al-Gezawy SM, El-Gezawy E, Azzaz AN. Honey as topical prophylaxis against radiochemotherapy-induced mucositis in head and neck cancer. J Laryngol Otol 2009;123:223–228. doi: 10.1017/S0022215108002478 . 18485252

[59] Bardy J, Molassiotis A, Ryder WD, Mais K, Sykes A, Yap B, et al. A double-blind, placebo-controlled, randomised trial of active Manuka honey and standard oral care for radiation-induced oral mucositis. Br J Oral Maxillofac Surg 2012;50:221–6. doi: 10.1016/j.bjoms.2011.03.005 . 21636188

[60] Charalambous M, Raftopoulos V, Paikousis L, Katodritis N, Lambrinou E, Vomvas D, et al. The effect of the use of thyme honey in minimizing radiation—induced oral mucositis in head and neck cancer patients: A randomized controlled trial. Eur J Oncol Nurs 2018;34:89–97. doi: 10.1016/j.ejon.2018.04.003 . 29784145

[61] Abbasi NM, Alami M, Sadr AB, Nikou FA, Erfan M, Azizian H. Allopurinol mouthwash for prevention or alleviation radiotherapy induced oral mucositis: A randomized, placebo-controlled trial. DARU J Pharm Sci 2007;15:227–30.

[62] Pitten FA, Kiefer T, Buth C, Doelken G, Kramer A. Do cancer patients with chemotherapy-induced leukopenia benefit from an antiseptic chlorhexidine-based oral rinse? A double-blind, block-randomized, controlled study. J Hosp Infect 2003;53(4):283–91. doi: 10.1053/jhin.2002.1391 . 12660125

[63] Schneider SB, Nishimura RD, Zimmerman RP, Tran L, Shiplacoff J, Tormey M, et al. Filgrastim (r-metHuG-CSF) and its potential use in the reduction of radiation-induced oropharyngeal mucositis: an interim look at a randomized, double-blind, placebo-controlled trial. Cytokines Cell Mol Ther 1999;5(3):175–80. . 10641576

[64] Su YB, Vickers AJ, Zelefsky MJ, Kraus DH, Shaha AR, et al. Double-blind, placebo-controlled, randomized trial of granulocyte-colony stimulating factor during postoperative radiotherapy for squamous head and neck cancer. Cancer J 2006;12(3):182–8. doi: 10.1097/00130404-200605000-00005 . 16803675

[65] Rahn R, Adamietz IA, Boettcher HD, Schaefer V, Reimer K, Fleischer W. Povidone-iodine to prevent mucositis in patients during antineoplastic radiochemotherapy. Dermatology 1997;195 Suppl 2:57–61. doi: 10.1159/000246032 . 9403257

[66] Sharma A, Rath GK, Chaudhary SP, Thakar A, Mohanti BK, Bahadur S. Lactobacillus brevis CD2 lozenges reduce radiation- and chemotherapy-induced mucositis in patients with head and neck cancer: a randomized double-blind placebo-controlled study. Eur J Cancer 2012;48:875–81. doi: 10.1016/j.ejca.2011.06.010 . 21741230

[67] Jiang C, Wang H, Xia C, Dong Q, Chen E, Qiu Y, et al. A randomized, double-blind, placebo-controlled trial of probiotics to reduce the severity of oral mucositis induced by chemoradiotherapy for patients with nasopharyngeal carcinoma. Cancer 2019;125:1081–90. doi: 10.1002/cncr.31907 . 30521105

[68] De Sanctis V, Belgioia L, Cante D, LA Porta MR, Caspiani O, Guarnaccia R, et al. Lactobacillus brevis CD2 for prevention of oral mucositis in patients with head and neck tumors: a multicentric randomized study. Anticancer Res 2019;39:1935–42. doi: 10.21873/anticanres.13303 . 30952736

[69] Mansourian A, Amanlou M, Shirazian S, Jahromi ZM, Amirian A. The effect of “Curcuma Longa” topical gel on radiation-induced oral mucositis in patients with head and neck cancer. Int J Radiat Res 2015:13(3): 269–274.

[70] Delavarian Z, Pakfetrat A, Ghazi A, Jaafari MR, Homaei Shandiz F, Dalirsani Z, et al. Oral administration of nanomicelle curcumin in the prevention of radiotherapy-induced mucositis in head and neck cancers. Spec Care Dentist 2019;39(2):166–72. doi: 10.1111/scd.12358 . 30761565

[71] Arun P, Sagayaraj A, Mohiyuddin SA, Santosh D. Role of turmeric extract in minimising mucositis in patients receiving radiotherapy for head and neck squamous cell cancer: a randomised, placebo-controlled trial. J Laryngol Otol 2020:1–6. doi: 10.1017/S0022215120000316 . 32029014

[72] Su CK, Mehta V, Ravikumar L, Shah R, Pinto H, Halpern J, et al. Phase II double-blind randomized study comparing oral aloe vera versus placebo to prevent radiation-related mucositis in patients with head-and-neck neoplasms. Int J Radiat Oncol Biol Phys 2004;60(1):171–7. doi: 10.1016/j.ijrobp.2004.02.012 . 15337553

[73] Puataweepong P, Dhanachai M, Dangprasert S, Sithatani C, Sawangsilp T, Narkwong L, et al. The efficacy of oral Aloe vera juice for radiation induced mucositis in head and neck cancer patients: a double-blind placebo-controlled study. Asian Biomedicine 2009;3:375–382.

[74] Yu YT, Deng JL, Jin XR, Zhang ZZ, Zhang XH, Zhou X. Effects of 9 oral care solutions on the prevention of oral mucositis: a network meta-analysis of randomized controlled trials. Medicine (Baltimore). 2020;99(16):e19661. doi: 10.1097/MD.0000000000019661 . 32311938 PMC7220734

[75] An W, Li S, Qin L. Role of honey in preventing radiation-induced oral mucositis: a meta-analysis of randomized controlled trials. Food Funct 2021;12(8):3352–3365. doi: 10.1039/d0fo02808h . 33900311

[76] Chirife J, Herszage L, Joseph A, Kohn ES. In vitro study of bacterial growth inhibition in concentrated sugar solutions: microbiological basis for the use of sugar in treating infected wounds. Antimicrob Agents Chemother 1983;23:766–773. doi: 10.1128/AAC.23.5.766 . 6870223 PMC184812

[77] Peng H, Chen BB, Chen L, Chen YP, Liu X, Tang LL, et al. A network meta-analysis in comparing prophylactic treatments of radiotherapy-induced oral mucositis for patients with head and neck cancers receiving radiotherapy. Oral Oncol. 2017;75:89–94. doi: 10.1016/j.oraloncology.2017.11.001 . 29224830

[78] Lai CC, Chen SY, Tu YK, Ding YW, Lin JJ. Effectiveness of low level laser therapy versus cryotherapy in cancer patients with oral mucositis: Systematic review and network meta-analysis. Crit Rev Oncol Hematol. 2021;160:103276. doi: 10.1016/j.critrevonc.2021.103276 . 33716203

[79] Ahmed S, Othman NH. Review of the medicinal effects of tualang honey and a comparison with manuka honey. Malays J Med Sci 2013;20(3):6–13. . 23966819 PMC3743976

[80] Karimi Z, Behnammoghadam M, Rafiei H, Abdi N, Zoladl M, Talebianpoor MS, et al. Impact of olive oil and honey on healing of diabetic foot: a randomized controlled trial. Clin Cosmet Investig Dermatol 2019;12:347–354. doi: 10.2147/CCID.S198577 . 31190942 PMC6516048

[81] Vandamme L, Heyneman A, Hoeksema H, Verbelen J, Monstrey S. Honey in modern wound care: a systematic review. Burns 2013;39:1514–1525. doi: 10.1016/j.burns.2013.06.014 . 23896128

[82] Chaitanya NC, Muthukrishnan A, Babu DBG, Kumari CS, Lakshmi MA, Palat G, et al. Role of vitamin E and vitamin A in oral mucositis induced by cancer chemo/radiotherapy–a meta‐analysis. J Clin Diagn Res 2017;11(5):ZE06‐ZE09. doi: 10.7860/JCDR/2017/26845.9905 . 28658926 PMC5483828

