# Supplementary figures and images for: Effects of various treatments for preventing oral mucositis in cancer patients: A network meta-analysis
Source: PLoS One. 2022 Dec 8;17(12):e0278102. doi: 10.1371/journal.pone.0278102 (PMC9731456; doi:10.1371/journal.pone.0278102)

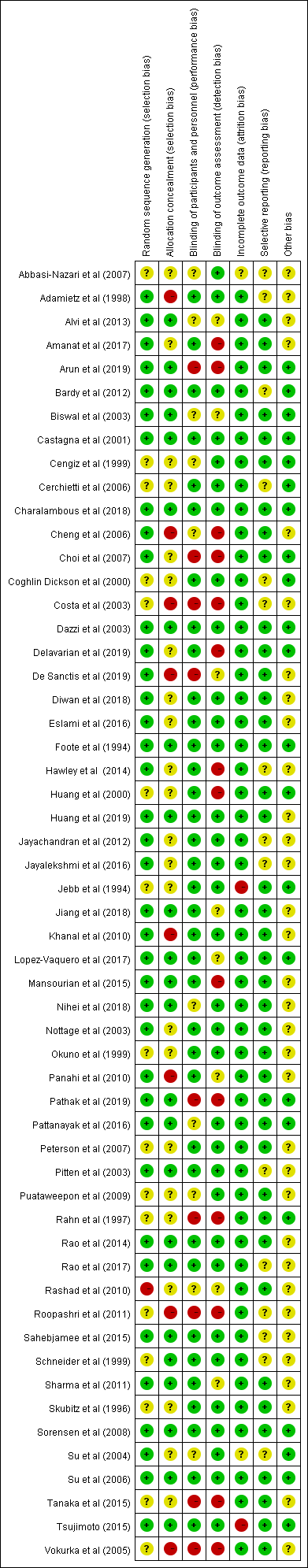

Supplement: S1 Fig — (TIF) [file pone.0278102.s001.tif]

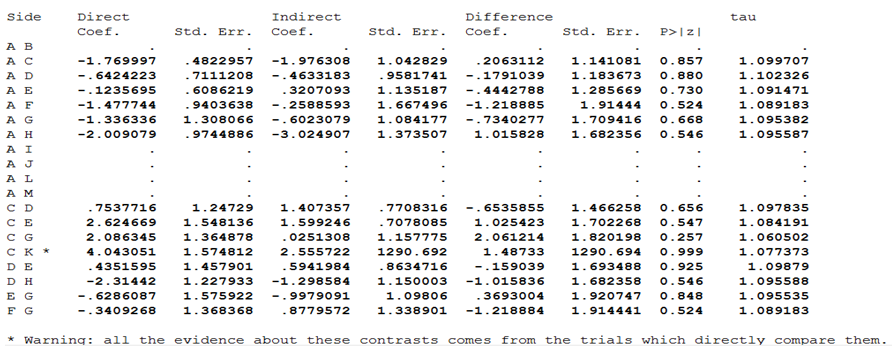

Supplement: S2 Fig — (Abbreviation: A, placebo; B, glutamine; C, honey; D, povidone-iodine; E, chlorhexidine; F, aloe; G, benzydamine; H, Curcumin; I, allopurinol; J, granulocyte-macrophage colony-stimulating factor; K, lignocaine; L, sucralfate; M, probiotics.). (TIF) [file pone.0278102.s002.tif]

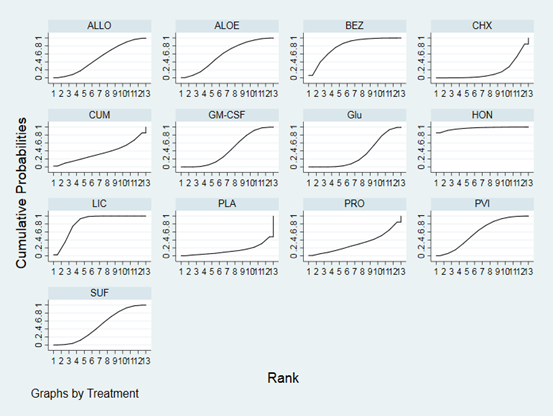

Supplement: S3 Fig — (Abbreviation: ALOE, aloe; ALLO, allopurinol; BEZ, benzydamine; CHX, chlorhexidine; CUM, Curcumin; Glu, glutamine; GM-CSF, granulocyte-macrophage colony-stimulating factor; HON, honey; LIC, lignocaine; PLA, placebo; PRO, probiotics; PVI, povidone-iodine; SUF, sucralfate.). (TIF) [file pone.0278102.s003.tif]

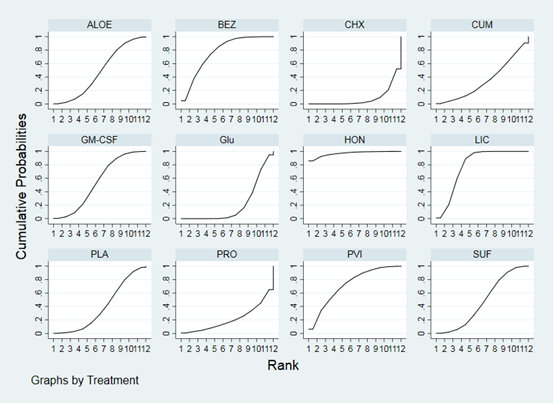

Supplement: S4 Fig — (Abbreviation: ALOE, aloe; ALLO, allopurinol; BEZ, benzydamine; CHX, chlorhexidine; CUM, Curcumin; Glu, glutamine; GM-CSF, granulocyte-macrophage colony-stimulating factor; HON, honey; LIC, lignocaine; PLA, placebo; PRO, probiotics; PVI, povidone-iodine; SUF, sucralfate.). (TIF) [file pone.0278102.s004.tif]
