# Supplementary material for: Effects of various treatments for preventing oral mucositis in cancer patients: A network meta-analysis
Source: PLoS One. 2022 Dec 8;17(12):e0278102. doi: 10.1371/journal.pone.0278102 (PMC9731456; doi:10.1371/journal.pone.0278102)
Supplement: S2 Table — (DOCX) [file pone.0278102.s007.docx]

**S2 Table.** Rank probability to be the best treatment (PrBest) by the incidence of radiotherapy-induced moderate-severe oral mucositis of each treatment in patients with cancer.

| Treatment | Pbest |
| --- | --- |
| Honey | 85.9% |
| Povidone-iodine | 6.3% |
| Benzydamine | 4.8% |
| Lignocaine | 1.0% |
| Probiotics | 0.06% |
| Curcumin | 0.05% |
| GM-CSF | 0.03% |
| Aloe | 0.02% |
| Placebo | 0.02% |
| Sucralfate | 0.01% |
| Glutamine | 0% |
| Chlorhexidine | 0% |
