# Supplementary material for: Effects of various treatments for preventing oral mucositis in cancer patients: A network meta-analysis
Source: PLoS One. 2022 Dec 8;17(12):e0278102. doi: 10.1371/journal.pone.0278102 (PMC9731456; doi:10.1371/journal.pone.0278102)
Supplement: S3 Table — (DOCX) [file pone.0278102.s008.docx]

**S3 Table.** Results of **the incidence of moderate-severe oral mucositis in patients with head and neck cancer;** results presented as constant odds ratios between all competing interventions with 95% confidence intervals. *Comparisons of treatments should be read from left to right. The rate ratio lower than 1 favors the top left treatment. The treatments have been sorted from left to right according to treatment ranking. Statistically significant differences between regimens are shown in bold with green background.

| **HON** |  |  |  |  |  |  |  |  |  |  |  |  |
| --- | --- | --- | --- | --- | --- | --- | --- | --- | --- | --- | --- | --- |
| 0.11 (0.00,2.50) | **LIC** |  |  |  |  |  |  |  |  |  |  |  |
| 0.10 (0.00,3.36) | 0.96 (0.20,4.55) | **BEZ** |  |  |  |  |  |  |  |  |  |  |
| 0.05 (0.00,1.37) | 0.43 (0.12,1.53) | 0.45 (0.06,3.33) | **PVI** |  |  |  |  |  |  |  |  |  |
| 0.04 (0.00,1.40) | 0.40 (0.08,2.00) | 0.42 (0.06,2.86) | 0.94 (0.12,7.27) | **ALOE** |  |  |  |  |  |  |  |  |
| 0.03 (0.00,1.08) | 0.30 (0.06,1.45) | 0.32 (0.04,2.60) | 0.71 (0.09,5.32) | 0.76 (0.12,4.72) | **ALLO** |  |  |  |  |  |  |  |
| **0.03 (0.00,0.83)** | **0.28 (0.08,0.97)** | 0.29 (0.05,1.81) | 0.66 (0.11,3.87) | 0.70 (0.10,4.96) | 0.93 (0.13,6.62) | **SUF** |  |  |  |  |  |  |
| **0.03 (0.00,0.67)** | **0.24 (0.10,0.59)** | 0.25 (0.04,1.49) | 0.55 (0.12,2.66) | 0.59 (0.09,3.72) | 0.78 (0.13,4.75) | 0.84 (0.18,3.85) | **GM-CSF** |  |  |  |  |  |
| 0.02 (0.00,1.49) | 0.14 (0.01,3.99) | 0.15 (0.00,5.86) | 0.33 (0.01,11.79) | 0.35 (0.01,14.27) | 0.47 (0.01,18.52) | 0.51 (0.01,17.65) | 0.60 (0.02,19.00) | **CUM** |  |  |  |  |
| **0.01 (0.00,0.95)** | 0.11 (0.01,2.33) | 0.12 (0.00,3.52) | 0.26 (0.01,7.02) | 0.28 (0.01,8.59) | 0.36 (0.01,11.13) | 0.39 (0.01,10.50) | 0.47 (0.02,11.21) | 0.78 (0.01,70.77) | **PRO** |  |  |  |
| **0.02 (0.00,0.36)** | **0.17 (0.07,0.39)** | **0.17 (0.03,0.97)** | 0.39 (0.08,1.81) | 0.41 (0.07,2.29) | 0.54 (0.09,3.13) | 0.59 (0.15,2.27) | 0.70 (0.20,2.40) | 1.16 (0.04,36.12) | 1.49 (0.06,35.21) | **Glu** |  |  |
| **0.01 (0.00,0.25)** | **0.07 (0.02,0.33)** | **0.08 (0.01,0.63)** | 0.17 (0.02,1.24) | 0.18 (0.02,1.61) | 0.24 (0.03,2.10) | 0.26 (0.05,1.32) | 0.31 (0.05,1.81) | 0.51 (0.01,19.80) | 0.65 (0.02,19.66) | 0.44 (0.08,2.44) | **CHX** |  |
| **0.00 (0.00,0.36)** | 0.03 (0.00,1.06) | 0.03 (0.00,1.51) | 0.06 (0.00,3.06) | 0.07 (0.00,3.67) | 0.09 (0.00,4.77) | 0.10 (0.00,4.59) | 0.12 (0.00,4.98) | 0.19 (0.00,26.91) | 0.25 (0.00,28.64) | 0.17 (0.00,7.09) | 0.38 (0.01,19.80) | **PLA** |

Abbreviation: ALOE, aloe; ALLO, allopurinol; BEZ, benzydamine; CHX, chlorhexidine; CUM, Curcumin; Glu, glutamine; GM-CSF, granulocyte-macrophage colony-stimulating factor; HON, honey; LIC, lignocaine; PLA, placebo; PRO, probiotics; PVI, povidone-iodine; SUF, sucralfate.
