# Supplementary material for: Effects of various treatments for preventing oral mucositis in cancer patients: A network meta-analysis
Source: PLoS One. 2022 Dec 8;17(12):e0278102. doi: 10.1371/journal.pone.0278102 (PMC9731456; doi:10.1371/journal.pone.0278102)
Supplement: S4 Table — (DOCX) [file pone.0278102.s009.docx]

**S4 Table.** Results of **the incidence of** **radiotherapy-induced moderate-severe oral mucositis in patients with cancer;** results presented as constant odds ratios between all competing interventions with 95% confidence intervals. *Comparisons of treatments should be read from left to right. The rate ratio lower than 1 favors the top left treatment. The treatments have been sorted from left to right according to treatment ranking. Statistically significant differences between regimens are shown in bold with green background.

| **HON** |  |  |  |  |  |  |  |  |  |  |  |
| --- | --- | --- | --- | --- | --- | --- | --- | --- | --- | --- | --- |
| 0.11 (0.01,1.73) | **LIC** |  |  |  |  |  |  |  |  |  |  |
| 0.11 (0.01,2.29) | 1.03 (0.29,3.73) | **BEZ** |  |  |  |  |  |  |  |  |  |
| 0.10 (0.00,2.66) | 0.96 (0.18,5.15) | 0.93 (0.11,7.72) | **PVI** |  |  |  |  |  |  |  |  |
| 0.05 (0.00,1.00) | 0.49 (0.18,1.33) | 0.47 (0.09,2.40) | 0.51 (0.07,3.61) | **GM-CSF** |  |  |  |  |  |  |  |
| **0.04 (0.00,0.87)** | 0.39 (0.11,1.45) | 0.38 (0.08,1.89) | 0.41 (0.05,3.46) | 0.81 (0.16,4.19) | **ALOE** |  |  |  |  |  |  |
| **0.04 (0.00,0.75)** | 0.38 (0.11,1.27) | 0.37 (0.07,1.81) | 0.40 (0.05,3.14) | 0.78 (0.16,3.67) | 0.96 (0.17,5.31) | **SUF** |  |  |  |  |  |
| **0.03 (0.00,0.67)** | 0.30 (0.08,1.04) | 0.29 (0.05,1.62) | 0.31 (0.04,2.52) | 0.61 (0.12,3.04) | 0.75 (0.17,3.33) | 0.78 (0.14,4.41) | **PLA** |  |  |  |  |
| **0.03 (0.00,0.78)** | 0.24 (0.03,1.72) | 0.24 (0.02,2.45) | 0.25 (0.02,3.35) | 0.50 (0.05,4.50) | 0.61 (0.06,6.47) | 0.64 (0.06,6.41) | 0.82 (0.08,8.39) | **CUM** |  |  |  |
| **0.01 (0.00,0.56)** | 0.11 (0.01,1.57) | 0.11 (0.01,2.05) | 0.12 (0.01,2.67) | 0.23 (0.01,3.87) | 0.28 (0.01,5.39) | 0.29 (0.02,5.40) | 0.37 (0.02,7.03) | 0.46 (0.02,12.37) | **PRO** |  |  |
| **0.02 (0.00,0.25)** | **0.16 (0.07,0.38)** | **0.16 (0.04,0.68)** | 0.17 (0.03,1.11) | 0.33 (0.09,1.20) | 0.41 (0.10,1.75) | 0.43 (0.12,1.46) | 0.55 (0.12,2.42) | 0.67 (0.08,5.65) | 1.46 (0.09,23.49) | **Glu** |  |
| **0.01 (0.00,0.17)** | **0.07 (0.02,0.31)** | **0.07 (0.01,0.46)** | **0.08 (0.01,0.71)** | **0.15 (0.03,0.87)** | 0.19 (0.03,1.26) | **0.20 (0.05,0.81)** | 0.25 (0.04,1.67) | 0.31 (0.03,3.47) | 0.67 (0.03,13.58) | 0.46 (0.10,2.22) | **CHX** |

Abbreviation: ALOE, aloe; ALLO, allopurinol; BEZ, benzydamine; CHX, chlorhexidine; CUM, Curcumin; Glu, glutamine; GM-CSF, granulocyte-macrophage colony-stimulating factor; HON, honey; LIC, lignocaine; PLA, placebo; PRO, probiotics; PVI, povidone-iodine; SUF, sucralfate.
