# Supplementary material for: Effects of various treatments for preventing oral mucositis in cancer patients: A network meta-analysis
Source: PLoS One. 2022 Dec 8;17(12):e0278102. doi: 10.1371/journal.pone.0278102 (PMC9731456; doi:10.1371/journal.pone.0278102)
Supplement: S5 Table — a. Excluding the study performed by Roopashri et al. b. Excluding the study performed by Roopashri and Costa et al. (DOCX) [file pone.0278102.s010.docx]

**S5 Table.** Results of **the incidence of moderate-severe oral mucositis**; results presented as constant odds ratios between all competing interventions with 95% confidence intervals. *Comparisons of treatments should be read from left to right. The rate ratio lower than 1 favors the top left treatment. The treatments have been sorted from left to right according to treatment ranking. Statistically significant differences between regimens are shown in bold with green background.

1. Excluding the study performed by Roopashri et al.

| **HON** |  |  |  |  |  |  |  |  |  |  |  |  |
| --- | --- | --- | --- | --- | --- | --- | --- | --- | --- | --- | --- | --- |
| 0.11 (0.00,2.79) | **LIC** |  |  |  |  |  |  |  |  |  |  |  |
| 0.11 (0.00,3.39) | 1.03 (0.31,3.36) | **BEZ** |  |  |  |  |  |  |  |  |  |  |
| 0.03 (0.00,1.24) | 0.31 (0.06,1.58) | 0.30 (0.04,2.12) | **ALLO** |  |  |  |  |  |  |  |  |  |
| 0.06 (0.00,1.88) | 0.56 (0.16,2.01) | 0.55 (0.10,3.05) | 1.83 (0.23,14.41) | **SUF** |  |  |  |  |  |  |  |  |
| 0.04 (0.00,1.61) | 0.41 (0.08,2.15) | 0.40 (0.06,2.49) | 1.34 (0.20,9.04) | 0.73 (0.09,5.72) | **ALOE** |  |  |  |  |  |  |  |
| 0.06 (0.00,2.29) | 0.54 (0.10,2.91) | 0.53 (0.07,4.14) | 1.77 (0.17,18.38) | 0.96 (0.12,7.95) | 1.32 (0.13,13.94) | **PRO** |  |  |  |  |  |  |
| 0.05 (0.00,1.57) | 0.43 (0.11,1.67) | 0.42 (0.07,2.54) | 1.39 (0.17,11.67) | 0.76 (0.12,4.88) | 1.04 (0.12,8.86) | 0.79 (0.09,6.83) | **PVI** |  |  |  |  |  |
| **0.01 (0.00,0.49)** | **0.07 (0.00,0.98)** | 0.07 (0.00,1.23) | 0.22 (0.01,5.05) | 0.12 (0.01,2.32) | 0.17 (0.01,3.82) | 0.12 (0.01,2.92) | 0.16 (0.01,3.17) | **PLA** |  |  |  |  |
| **0.03 (0.00,0.89)** | **0.30 (0.14,0.61)** | 0.29 (0.07,1.16) | 0.96 (0.16,5.76) | 0.52 (0.12,2.24) | 0.72 (0.12,4.38) | 0.54 (0.09,3.40) | 0.69 (0.15,3.23) | 4.35 (0.27,69.22) | **GM-CSF** |  |  |  |
| 0.04 (0.00,1.42) | 0.36 (0.08,1.71) | 0.35 (0.05,2.48) | 1.17 (0.12,11.17) | 0.64 (0.09,4.72) | 0.87 (0.09,8.47) | 0.66 (0.07,6.54) | 0.84 (0.11,6.63) | 5.29 (0.24,116.28) | 1.22 (0.22,6.77) | **CUM** |  |  |
| **0.01 (0.00,0.38)** | **0.10 (0.02,0.47)** | **0.09 (0.01,0.67)** | 0.31 (0.03,3.03) | **0.17 (0.03,0.93)** | 0.23 (0.02,2.28) | 0.18 (0.02,1.78) | 0.22 (0.03,1.81) | 1.41 (0.06,31.43) | 0.32 (0.06,1.86) | 0.27 (0.03,2.46) | **CHX** |  |
| **0.02 (0.00,0.41)** | **0.16 (0.07,0.39)** | **0.16 (0.04,0.63)** | 0.53 (0.09,3.29) | 0.29 (0.07,1.17) | 0.40 (0.07,2.32) | 0.30 (0.05,2.01) | 0.38 (0.08,1.93) | 2.41 (0.15,40.09) | 0.56 (0.18,1.72) | 0.46 (0.08,2.71) | 1.72 (0.29,10.08) | **Glu** |

1. Excluding the study performed by Roopashri and Costa et al.

| **HON** |  |  |  |  |  |  |  |  |  |  |  |  |
| --- | --- | --- | --- | --- | --- | --- | --- | --- | --- | --- | --- | --- |
| 0.10 (0.00,2.58) | **LIC** |  |  |  |  |  |  |  |  |  |  |  |
| 0.15 (0.00,4.60) | 1.47 (0.42,5.10) | **BEZ** |  |  |  |  |  |  |  |  |  |  |
| 0.03 (0.00,1.18) | 0.32 (0.06,1.60) | 0.22 (0.03,1.54) | **ALLO** |  |  |  |  |  |  |  |  |  |
| 0.06 (0.00,1.77) | 0.58 (0.16,2.01) | 0.39 (0.07,2.24) | 1.81 (0.24,13.84) | **SUF** |  |  |  |  |  |  |  |  |
| 0.05 (0.00,1.65) | 0.46 (0.09,2.34) | 0.31 (0.05,1.93) | 1.43 (0.22,9.47) | 0.79 (0.10,6.08) | **ALOE** |  |  |  |  |  |  |  |
| 0.06 (0.00,2.09) | 0.54 (0.10,2.83) | 0.37 (0.05,2.91) | 1.70 (0.17,17.16) | 0.94 (0.12,7.54) | 1.19 (0.12,12.16) | **PRO** |  |  |  |  |  |  |
| 0.04 (0.00,1.44) | 0.43 (0.11,1.63) | 0.29 (0.05,1.81) | 1.35 (0.17,10.94) | 0.75 (0.12,4.64) | 0.94 (0.11,7.76) | 0.79 (0.09,6.63) | **PVI** |  |  |  |  |  |
| **0.01 (0.00,0.45)** | **0.07 (0.00,0.96)** | **0.05 (0.00,0.86)** | 0.21 (0.01,4.76) | 0.12 (0.01,2.22) | 0.15 (0.01,3.36) | 0.13 (0.01,2.86) | 0.16 (0.01,3.09) | **PLA** |  |  |  |  |
| **0.03 (0.00,0.83)** | **0.30 (0.14,0.61)** | **0.20 (0.05,0.85)** | 0.93 (0.16,5.47) | 0.52 (0.12,2.16) | 0.65 (0.11,3.89) | 0.55 (0.09,3.34) | 0.69 (0.15,3.16) | 4.37 (0.28,68.15) | **GM-CSF** |  |  |  |
| 0.04 (0.00,1.32) | 0.36 (0.08,1.68) | 0.25 (0.03,1.77) | 1.14 (0.12,10.53) | 0.63 (0.09,4.51) | 0.79 (0.08,7.46) | 0.67 (0.07,6.38) | 0.84 (0.11,6.44) | 5.31 (0.25,113.66) | 1.22 (0.22,6.61) | **CUM** |  |  |
| **0.01 (0.00,0.35)** | **0.10 (0.02,0.46)** | **0.07 (0.01,0.48)** | 0.30 (0.03,2.87) | **0.17 (0.03,0.90)** | 0.21 (0.02,2.02) | 0.18 (0.02,1.74) | 0.22 (0.03,1.77) | 1.42 (0.07,30.86) | 0.32 (0.06,1.82) | 0.27 (0.03,2.39) | **CHX** |  |
| **0.02 (0.00,0.39)** | **0.17 (0.07,0.41)** | **0.12 (0.03,0.48)** | 0.54 (0.09,3.25) | 0.30 (0.08,1.18) | 0.38 (0.07,2.14) | 0.32 (0.05,2.06) | 0.40 (0.08,1.96) | 2.52 (0.16,40.97) | 0.58 (0.19,1.76) | 0.47 (0.08,2.74) | 1.78 (0.31,10.22) | **Glu** |

Abbreviation: ALOE, aloe; ALLO, allopurinol; BEZ, benzydamine; CHX, chlorhexidine; CUM, Curcumin; Glu, glutamine; GM-CSF, granulocyte-macrophage colony-stimulating factor; HON, honey; LIC, lignocaine; PLA, placebo; PRO, probiotics; PVI, povidone-iodine; SUF, sucralfate.
